# Peer replication: A new tier of science built on reproducibility

**DOI:** 10.1038/s44319-026-00705-8

**Published:** 2026-02-09

**Authors:** Samuel J Lord, Arthur Charles-Orszag, Kristen Skruber, R Dyche Mullins, Anders Rehfeld

**Affiliations:** 1 UCSF & HHMI, 600 16th St, Genentech Hall N314, San Francisco, CA 94143 USA; 2 Department of Microbiology and Molecular Genetics, University of California Davis, Davis, CA USA; 3 Department of Biology, Gonzaga University, Spokane, WA 99258 USA; 4 Department of Growth and Reproduction, Copenhagen University Hospital - Rigshospitalet, Copenhagen, Denmark; 5 Department of Cellular and Molecular Medicine, Faculty of Health Sciences, University of Copenhagen, Copenhagen, Denmark; 6 Department of Clinical Biochemistry, North Zealand Hospital, Hillerød, Denmark

**Keywords:** Science Policy & Publishing

## Abstract

To address the replication crisis and instill confidence in the scientific literature, we introduce a new framework for evaluating scientific manuscripts. “Peer replication” would be an alternative or augmentation to peer review and elevate peer-replicable works to a higher tier of publication.

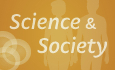

Scientific publishing faces at least three major challenges: there are too many papers for a single human to read, even in highly specialized fields; most published research is unlikely to be reproducible (Errington et al, 2021; Drude et al, 2021), either because the original experiments are flawed or because the methods are not sufficiently described; and peer review has proven burdensome to both authors and reviewers and yet ineffective at screening out low-quality science or outright fraud (Schroter et al, 2008).

While peer review usually increases the quality of manuscripts, the process is onerous and has limited benefit: often, there is little difference in the core elements of a preprint and its final published form (Carneiro et al, 2020). One reason may be that peer review evaluates a manuscript based on the opinions of very few people at the beginning of its lifetime rather than its ability to stand up against the key aspects of the scientific method: orthogonal testing and replication. Moreover, the current publishing system introduces distorted incentives: authors are motivated to present splashy but not necessarily robust findings, and reviewers are often motivated to demand superfluous controls or entirely new experiments. To address these shortcomings, we introduce an alternative to traditional peer review, termed “peer replication” (Fig. 1), whereby fellow researchers reproduce the key findings of a manuscript to ensure rigorous and trustworthy science along with more detailed reporting.

**Figure 1:**
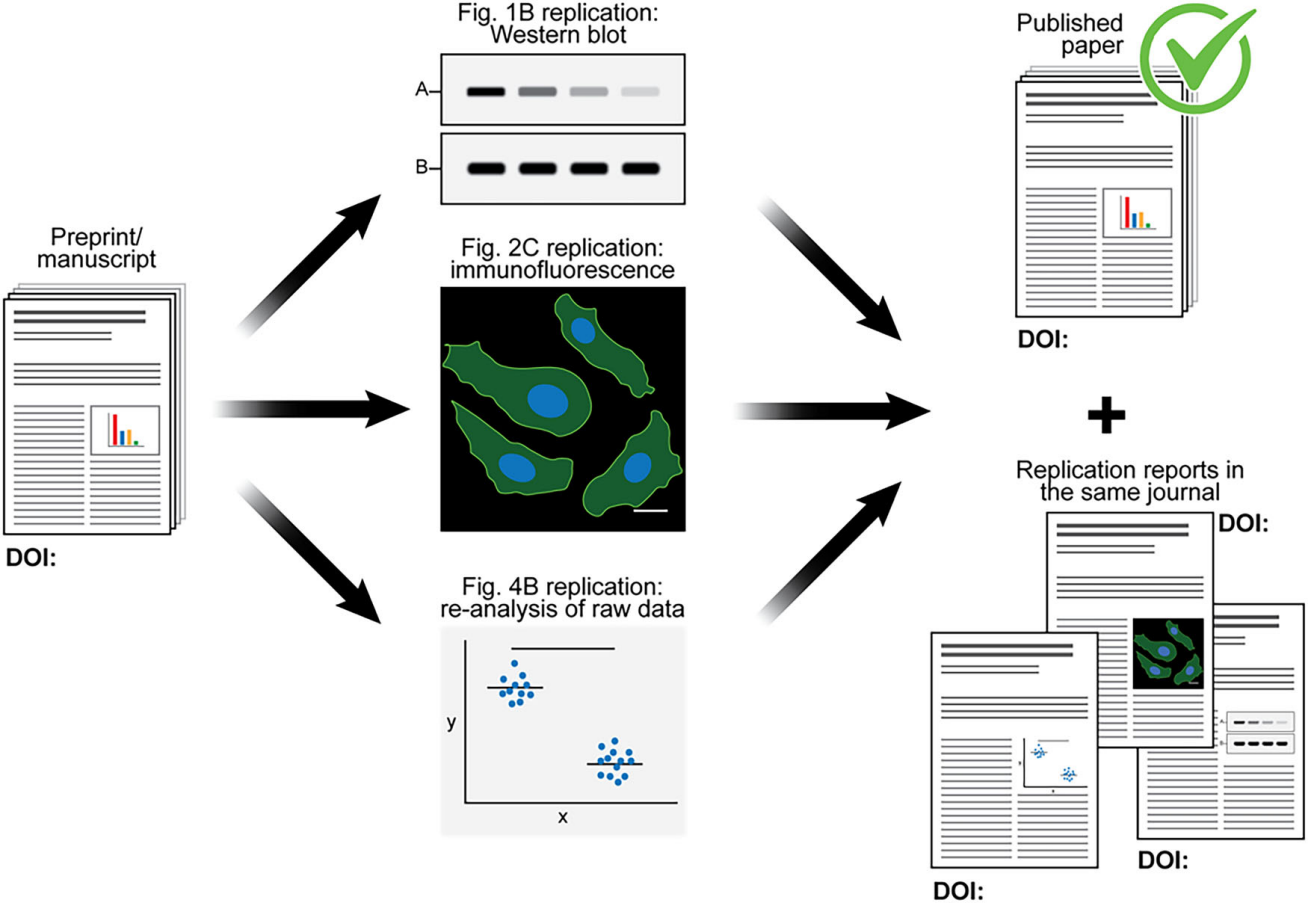
A scheme summarizing an example Peer Replication process. *Left*: Manuscript is selected for peer replication by the editor, and peer replicators are invited to replicate key experiments or analyses. *Center*: Key experiments and analyses are replicated by peers. *Right*: Peer Replication Reports are published in the journal together with the published original paper. The journal would label the published paper as peer-replicated.

“We introduce an alternative to traditional peer review, termed “peer replication” whereby fellow researchers reproduce the key findings of a manuscript …”

Schemes similar to peer replication have already been implemented to some extent. For example, the Institute for Replication arranges “workshops to replicate selected papers within economics and political sciences, and publish replication reports in meta-papers (Brodeur et al, 2023). Epic Research arranges clinical trials with multiple independent groups running the experiment in parallel, and some complex datasets have been independently analyzed by multiple teams (Wagenmakers et al, 2022). For more than a century, the journal *Organic Syntheses* has ensured that the instructions it publishes are easy to follow and reproducible by repeating the synthesis in an editor’s laboratory (Kamm et al, 1921). While synthetic chemistry is especially amenable to peer replication, many techniques in other fields—Western blots, cell-signaling assays, chemical analysis, flow cytometry, mass spectrometry, microscopy, transient transfections, electrophysiology, reanalysis of raw data, and so on—could be subjected to the same approach.

Multiple other replication initiatives have also tried to address the problem of reproducibility in science (Heyard et al, 2025), including topic-specific approaches such as the *The Reproducibility Project: Cancer Biology* from the Center for Open Science, as well as geographically-oriented projects such as *The Brazilian Reproducibility Initiative*. In contrast to most prior initiatives, our peer-replication model would evaluate replicability during the publication process at any journal as an integrated part of their evaluation. By providing citable Peer Replication Reports alongside the published paper, readers will be able to evaluate any replicated manuscript without needing to test the results on their own or relying on others to eventually report replication attempts elsewhere.

“In contrast to most prior initiatives, our peer-replication model would evaluate replicability during the publication process at any journal as an integrated part of their evaluation.”

Peer replication could thereby supplant or augment traditional peer review and become a third tier of publication: preprints, peer-reviewed papers, and peer-replicated papers. The process we propose is only one potential model. Journals could choose to implement peer replication in different ways to align with their publishing model and achieve the goal of elevating reproducibility as a key factor that is communicated to the readers.

## A proposed process of peer replication

1. A journal editor selects a manuscript for peer replication. Authors would opt-in to the peer-replication track during submission.
2. The editor approaches researchers to serve as “peer replicators” on the manuscript, which means that they are invited to replicate selected key experiments or analyses in the manuscript. In consultation with the editor, peer replicators decide how to do so and can involve relevant co-authors. The replication plan would then be pre-registered.
3. After consultation with the editor, the authors of the original manuscript can share reagents—especially those that may not be commercially available—with peer replicators, assist with the experimental design, and allow access to specialized equipment or custom software if necessary for performing the replication experiments. The editor’s role would be to maintain independence between the labs while promoting expediency.
4. Journals may choose to recruit peer reviewers separately or have the peer replicators themselves review the manuscript.
5. Once a replication attempt is completed, the results are published alongside the original manuscript in the form of a short and citable “Peer Replication Report” with its own DOI.

Some replication attempts may fail, but a failure to replicate does not necessarily negate the original findings. For instance, a replication attempt may fail due to important variables not accounted for in the methods. In that case, the editor could work with the authors and peer replicators to track down the problem, strengthening the methods section and technique for future experiments. Furthermore, editors may choose to recruit multiple independent replicators for the same experiments, just like they solicit multiple reviews. Importantly, the Peer Replication Reports should be published regardless of the outcome to ensure transparency and maintain an unbiased incentive for replicators to accept the task. Journals will need to develop policies for dealing with submissions when the replication attempts failed. Ultimately, readers would be empowered to decide how to evaluate a paper’s original findings along the well-documented Peer Replication Reports regardless of the outcome of the replication attempts.

## Incentives aligned

The incentive for a researcher to volunteer their lab’s time and resources to try to reproduce someone else’s experiment would be simple: credit in the form of a citable published Peer Replication Report in the same journal as the original manuscript. Unlike peer review, the referees will receive compensation for their work in the form of citations and another publication on their CV. To minimize the burden, peer replication would need to be limited to simple key experiments using assays the replicating lab already uses.

“Unlike peer review, the referees will receive compensation for their work in the form of citations and another publication on their CV.”

Authors will volunteer to subject their work to peer replication for multiple reasons. Above all, most scientists want to publish real findings, and witnessing a colleague repeat your results is a reward in itself. Additionally, the process would be easier than traditional peer review, which puts a huge burden on authors to perform additional experiments and defend their work against criticism. Peer replication turns the process on its head, with the referees doing the work of validating the manuscript’s findings.

## Ancillary benefits

A successful replication is clearly superior to reviews based on reading alone, but the peer replication process would introduce additional benefits. Most importantly, it would require that adequate protocols are provided to the scientific community if the findings have any chance of a successful replication. In addition, peer replication would address the issue that replication studies and negative results have generally been difficult to publish (National Academies of Sciences, 2019).

“Peer replication transforms the adversarial process of peer review into a cooperation among colleagues to improve scientific rigor.”

Furthermore, peer replication transforms the adversarial process of peer review into a cooperation among colleagues to improve scientific rigor. Another set of eyes and brains on an experiment could introduce additional controls or alternative experimental approaches that would bolster and even expand the original finding. This approach also encourages sharing experimental procedures among labs in a manner that can foster future collaborations, inspire novel approaches, and train students and postdocs in a wider range of techniques. Too often, valuable hands-on knowledge is sequestered in individual labs; peer replication would offer an avenue to disseminate those skills.

“Too often, valuable hands-on knowledge is sequestered in individual labs; peer replication would offer an avenue to disseminate those skills.”

Finally, peer replication would reduce fraud. It would be nearly impossible for a researcher to pass off fabricated data or manipulated images as real if other researchers will be tasked with actually reproducing the experimental results.

## Challenges

The major hurdles to implementing Peer Replication include: finding researchers willing to undertake replications; not all experiments can be readily repeated in a reasonable amount of time; the added time and complexity to get a paper published; the cost to perform the replications; and publishing Peer Replication Reports in a manner that doesn’t reduce a journal’s Impact Factor. These five major hurdles are discussed in greater depth below.

Scientific journals are experiencing decreased reviewer acceptance rates, known as “reviewer fatigue” (Routledge et al, 2025). A potential solution could be changing the incentive structure of the publishing system. Unlike peer review, the referees will receive compensation for their work in the form of another publication to include on their CV. To minimize the burden, replications would focus on simple key experiments using assays the replicating lab already uses. Importantly, and unlike peer review, which is most often undertaken by a single invited researcher, peer replication can involve collaborators and students, who will become co-authors on the Peer Replication Report. Most labs would be able to find the time and funds to run a few simple experiments and immediately get authorship on a paper, especially if existing assays and equipment can be reused. This may be particularly appealing to research groups with early-career researchers who would benefit from learning new techniques and building their list of publications. Peer replication would thus enable researchers who are normally not invited as peer reviewers, for instance, younger scientists and/or scientists from emerging regions, to contribute to the process while at the same time providing them with an opportunity to get a first-authored publication.

Peer reviewers today are already estimated to spend more than 100 million hours per year collectively (Dance, 2023). Using even a tiny part of this time on attempting to replicate key findings rather than simply evaluating the text and data of a manuscript would undoubtedly benefit science. However, there would never be enough time or resources to repeat even all the simplest experiments that are published in thousands of journals. Editors, and the availability of peer replicators, would ultimately dictate which papers get selected for peer replication.

Of course, many experiments will not be feasible to attempt a one-to-one replication. But most papers will have some core aspects that can be replicated. While it would not be possible to repeat a clinical trial, referees could run independent analyses on the raw data (Wagenmakers et al, 2022). Similarly, a peer may not be able to build a new microscope, but they may be able to bring their own samples to the author’s lab and perform their own imaging to test the equipment (Millett-Sikking and York, 2019). Editors—in consultation with authors and referees—will determine the set of key experiments that will undergo replication, balancing rigor and feasibility. Ultimately, some fields will be more amenable to this process and may provide good testing grounds for peer replication. While this may leave some of the most complex experiments unreplicated, it will be up to readers to judge a paper as a whole. It should also be noted that modern clinical trials generally meet much higher standards for pre-registration, reporting, and sample sizes than the typical research article found in most journals. In fields where replication is especially challenging, rigor could be achieved in other ways.

Time is precious, and even small experiments will take some effort to repeat. Journals may thus choose to simply publish peer-reviewed manuscripts with an “Awaiting peer replication” label, which would be no slower than the current system. Regardless, editors will need to select only key experiments for replication, minimizing the time and effort burden on everyone involved. Many experiments could be replicated in a few weeks at most. Peer review is already a slow process (Royle, 2020), often taking months and multiple rounds of revision, and in many cases ends in rejection. With the rapid adoption of preprints across many domains of science, new results can be disseminated quickly, so the need to accelerate the publication of the final form is alleviated. If, in practice, peer replication does take more time than traditional peer review, it should nonetheless increase the strength of the findings enough that it will result in a net reduction in lost time due to irreproducible experiments that can hamper a field and stall progress.

Science funding is also precious, and labs will need to find ways to pay for replications. Ideally, funding agencies would create small on-demand grants to cover the costs of peer-replication experiments. Institutes or funding agencies could require that the work they fund be replicated and allocate earmarked funding for such studies. Ultimately, funding agencies are the prime movers in academic research. If they demand reproducible science, incentives will naturally realign closer to that standard, and everyone would be further encouraged to participate in peer replication.

Alternatively, publication fees paid by the authors could help fund the replication, in which case the editor would need to carefully select only low-cost experiments. Some labs will be able to find the time and funds to run a few simple experiments and immediately get authorship on a paper, especially if existing assays and equipment can be reused.

Some journals may be resistant to experimenting with novel models of evaluation, as they fear this could decrease their Impact Factor. We therefore recommend that the published Peer Replication Reports are not indexed as “articles” by Clarivate and are thus omitted from the denominator when calculating the Impact Factor, similar to what is already done for editorial material and letters. Alternatively, Peer Replication Reports could be bundled with the original paper, as is done with public peer review.

## Moving forward

To implement peer replication, we need to include additional stakeholders in the publication process, including researchers, institutions, funding agencies, and journals. Forward-thinking organizations such as non-profit open-access journals, preprint servers, or research institutes would be an integral part of trialing and implementing peer replication.

In the meantime, authors can and should add replication robustness even without external journal-driven initiatives. First and foremost, researchers should repeat their experiments multiple times, collect data and conduct analysis blinded, and perform statistics on biological replicates (Lord et al, 2020; Lazic, 2010). Researchers may also seek their own form of peer replication by recruiting collaborators and colleagues to perform replications of key experiments and adding them as co-authors on the subsequent manuscript. Ultimately, authors, reviewers, editors, funders, and institutions celebrating work that has been replicated will drive scientific norms towards replication robustness. While peer replication has the promise to help align incentives and cure the replication crisis, we can already each contribute to robust science and reproducibility.

## Supplementary information

Peer Review File
