# Supplementary material for: Peer replication: A new tier of science built on reproducibility
Source: EMBO Rep. 2026 Feb 9;27(5):1093–7. doi: 10.1038/s44319-026-00705-8 (PMC12979767; doi:10.1038/s44319-026-00705-8)
Supplement: Supplementary file 1 — Peer Review File [file 44319_2026_705_MOESM1_ESM.pdf]

EMBO reports

Peer Replication

Anders Rehfeld, Samuel Lord, Arthur Charles-Orszag, Kristen Skruber, and R. Dyche Mullins

Corresponding author(s): Anders Rehfeld (rehfeld@sund.ku.dk) , Samuel Lord (Samuel.Lord@ucsf.edu)

Review Timeline:

Submission Date:	29th Dec 25
Editorial Decision:	8th Jan 26
Revision Received:	19th Jan 26
Accepted:	26th Jan 26

Editor: Holger Breithaupt

Transaction Report: This article was editorially reviewed.
